# Spatio-temporal dataset of building occupants

**DOI:** 10.1016/j.dib.2019.104598

**Published:** 2019-10-10

**Authors:** Muhammad Arslan, Christophe Cruz, Dominique Ginhac

**Affiliations:** a Laboratoire d’Informatique de Bourgogne (LIB) – EA 7534, Univ. Bourgogne Franche-Comte, Dijon 21000, France; b Laboratoire Imagerie et Vision Artificielle (ImViA) – EA 7535, Univ. Bourgogne Franche-Comte, Dijon 21000, France

**Keywords:** Buildings, BLE beacons, Indoor environments, Occupants, Locations, Trajectories

## Abstract

The paper presents spatio-temporal dataset of building occupants captured using 200 Bluetooth Low Energy (BLE) beacons installed on different locations in two buildings. It contains 8426 data points of 11 building occupants collected with a sampling rate of 5 seconds during different times in a 12 days' interval. Each spatio-temporal data point comprises location and time components correspond to a building location which can be visualized using an OpenStreetMap (OSM) file of a building.

**Table:** Specifications Table

Subject area	Computer Science Applications
More specific subject area	Sensory systems, Occupant behaviors
Type of data	Comma Separated File (CSV), table and figure
How data was acquired	BLE beacons, Android-based mobile application
Data format	Raw
Experimental factors	The null location values were deleted from a dataset.
Experimental features	Around 200 BLE beacons were installed in a building within a radius of 4 - 5 m. Using Android-based mobile application, location of building users with timestamps were collected.
Data source location	IUT Dijon (21000), France
Data accessibility	1) Dataset is available with this article. 2) Mendely Link: https://doi.org/10.17632/5hhxtzj5gm.1
Related research article	M. Arslan, C. Cruz and D. Ginhac, Visualizing intrusions in dynamic building environments for worker safety, Safety Science 120 (2019) 428–446, https://doi.org/10.1016/j.ssci.2019.07.020

**Table:** 

**Value of the Data** • The obtained spatio-temporal dataset can be used as benchmark datasets to test the accuracy in perceiving the occupant movements for different built environment applications. • As spatio-temporal data holds multifaceted characteristics such as position, direction, speed, change in direction and distance travelled information of moving objects. By analyzing these characteristics, occupants' activities and their behavioral patterns can be inferred. • Dataset can be used by researchers for performing advanced spatio-temporal data analysis. State-of-the-art 3D visualization tools such as Building Information Modeling (BIM) can be used to study the occupant movements in building environment context.

## 1 Data

Two data files are provided for understanding spatio-temporal movements [1,2] of building occupants. The 1st data file contains five fields (see Table 1) which are; 1. userID (a random identity of an occupant), 2. DateTime (a timestamp when a data point is recorded), 3. Building floor (the level of building from where a data point is recorded), 4. Longitude and 5. Latitude values on a geographical scale. Also, an OSM file (named: IUT_Venue.OSM) of building from where the spatio-temporal data is recorded is also included. The file structure and the contents of an OSM file are universal and widely documented in the existing literature.

**Table 1:** No. of records per occupant in collected spatio-temporal dataset.

Building users	Days												Grand total
	1	2	3	4	5	6	7	8	9	10	11	12	
User A				50									50
User B					236	6			29	46			317
User C				300									300
User D				365									365
User E		1368		235									1603
User F				6									6
User G				14									14
User H				123	17	7	1653	96	15				1911
User I	290	175	410	123	176	192	28				201	229	1824
User J				1400	500								1900
User K				119	17								136
**Grand Total**	**290**	**1543**	**410**	**2735**	**946**	**205**	**1681**	**96**	**44**	**46**	**201**	**229**	**8426**

## 2 Experimental design, materials, and methods

**Fig. 1:**
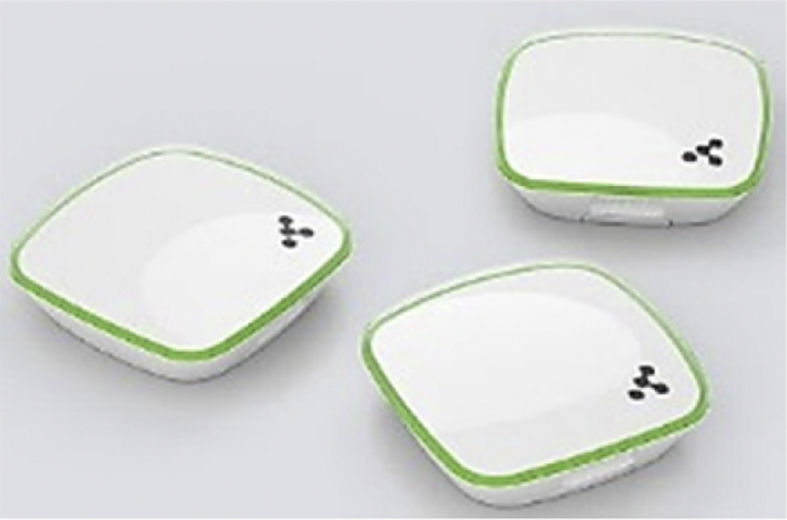
BLE beacons by Kontakt.io used for data collection.

**Fig. 2:**
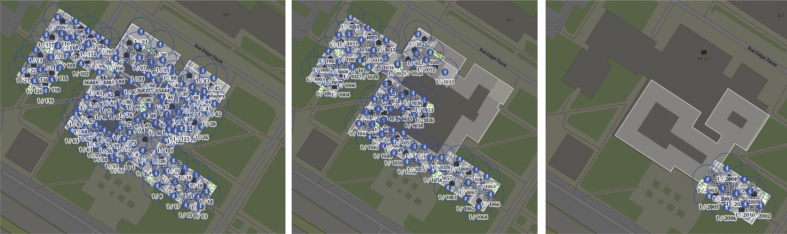
Mounting BLE beacons on different floors in a building [3,4].

**Fig. 3:**
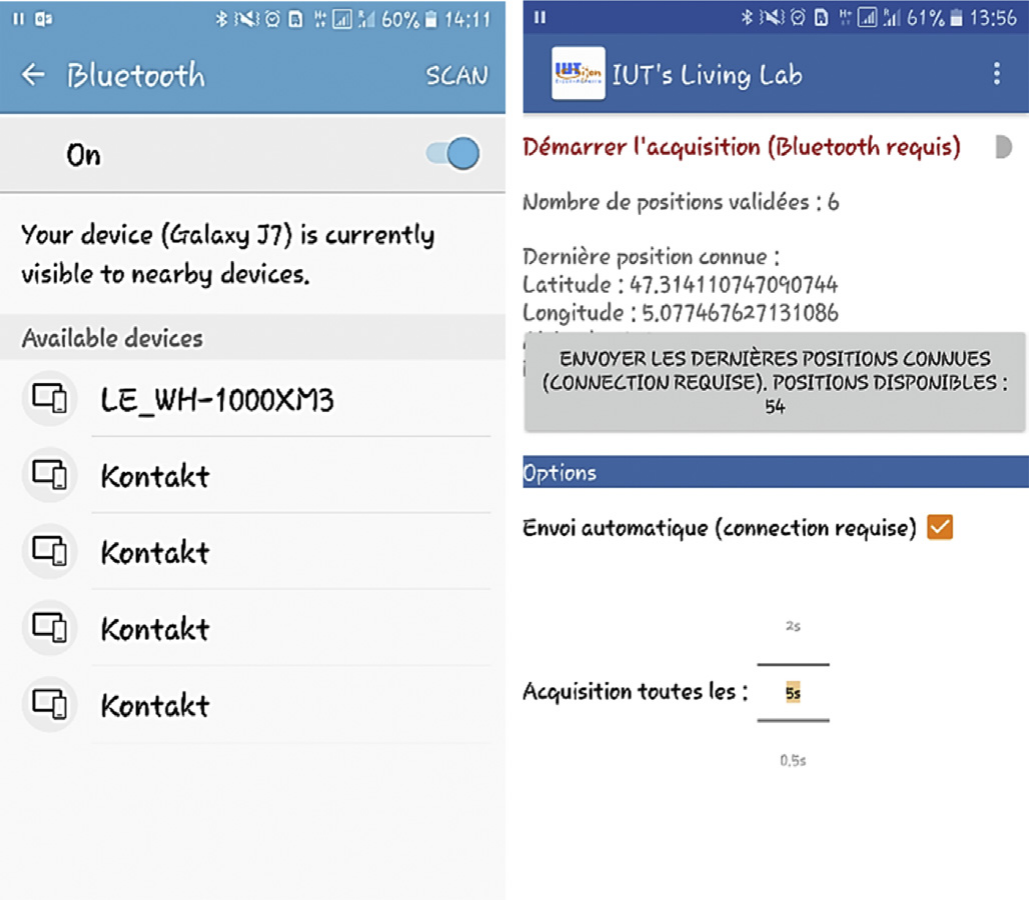
Mobile application to collect spatio-temporal data [3,4].

**Fig. 4:**
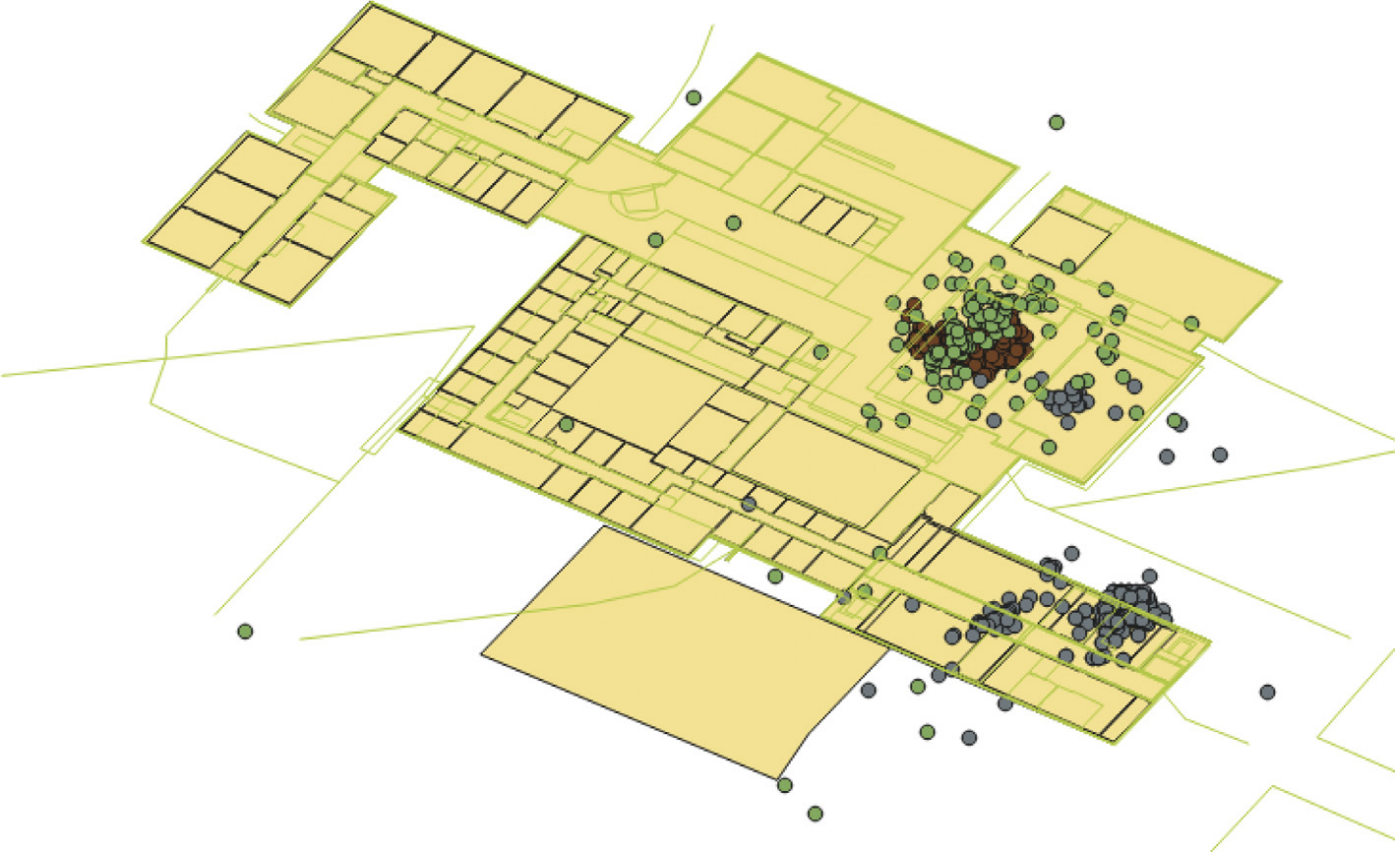
A sample data of three different occupants' locations detected using BLE beacons in three different colors.

Low cost 200 BLE beacons (see Fig. 1) were mounted on different locations in a building (see Fig. 2) to acquire occupant locations. Each beacon was configured to broadcast a Bluetooth signal within in radius of 4–5 m. To estimate the occupant locations, an Android-based mobile application was developed (see Fig. 3). As the application is launched in an occupant's mobile device, it detects the neighboring mounted beacons. Based on the received signal strength, it selects the best three beacons in its range and preforms geo-localization technique to estimate the occupant location and generates a longitude and latitude pair value. The process of generating location coordinates is achieved after mapping the collected location coordinates with the stored spatial information residing in a database (see Fig. 4). The sampling interval was kept 5 seconds to collect the data.

## 3 Discussion

**Fig. 5:**
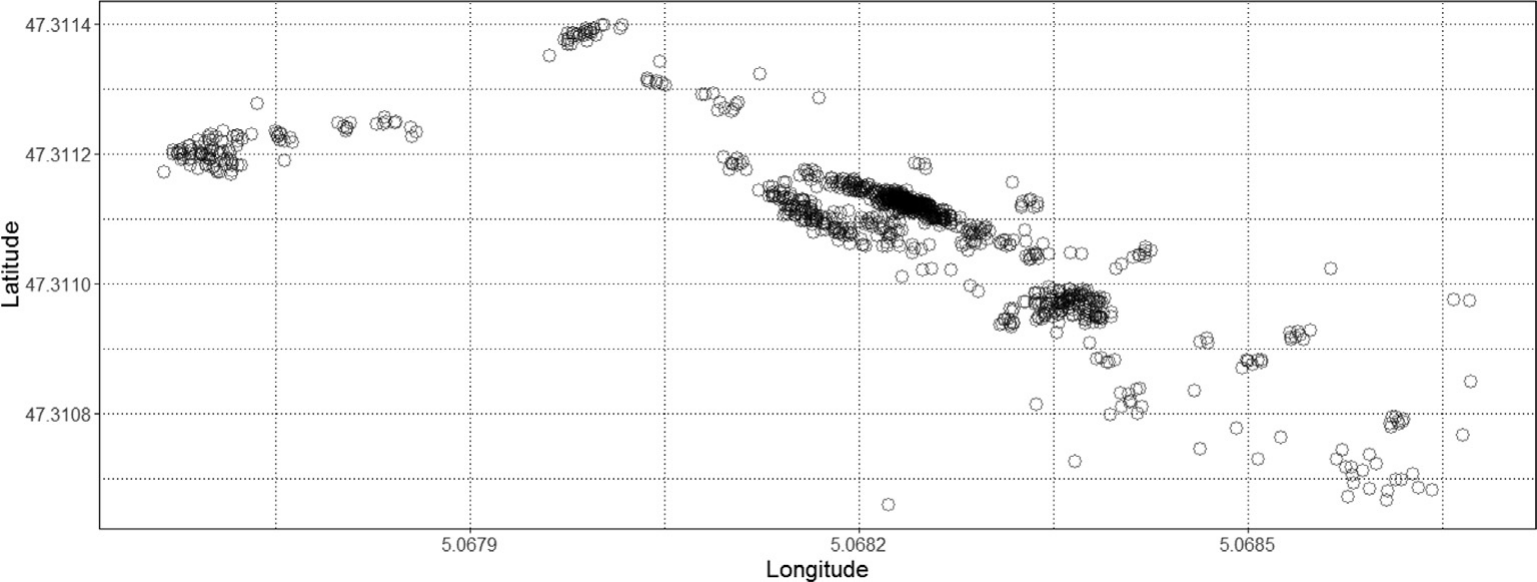
Raw spatio-temporal data of a user.

**Fig. 6:**
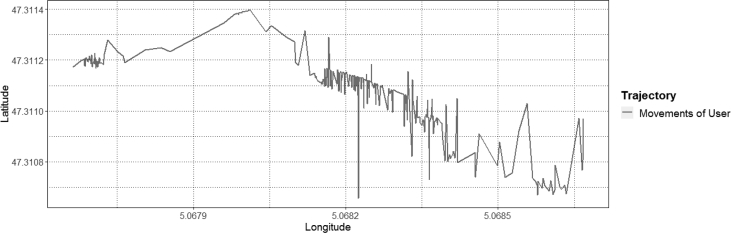
Processed spatio-temporal trajectory from the provided dataset of a user.

**Fig. 7:**
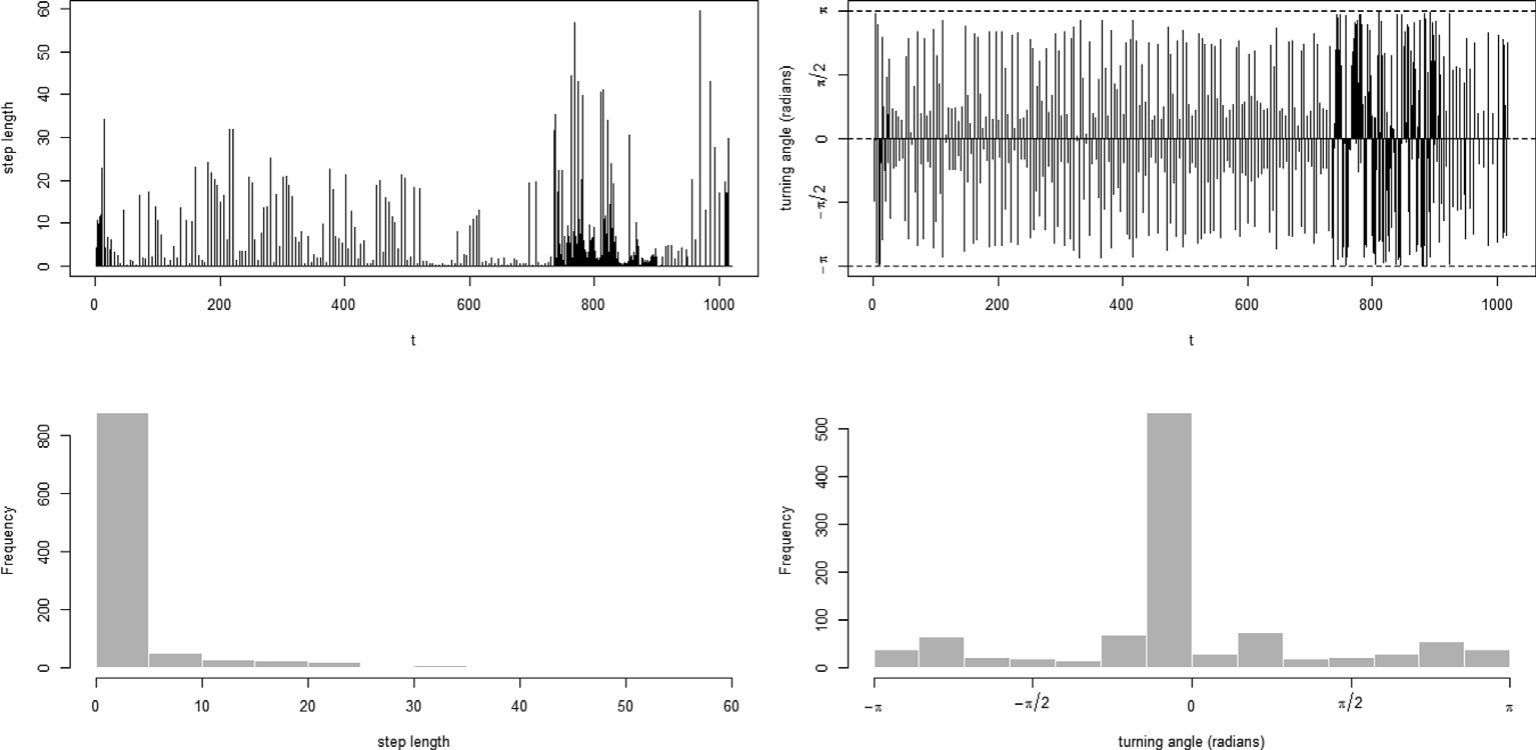
Time series plots (top row) and histograms (bottom row) of the step lengths and turning angles of a user.

The described dataset presented above has been utilized in developing applications for monitoring worker safety by analyzing their movements in real-time [3,4]. As the collected spatio-temporal data consist of ordered sequences of discrete-time triples in the form of 〈latitude, longitude, timestamp〉 include a timestamp (i.e. the position of an occupant on a timescale) mapped with the position of an occupant in the geographical coordinate system (see Fig. 5). The multifaceted characteristics such as the speed of an occupant (i.e. step length), direction to which an occupant travels (also called as turning angle), and distance covered by an occupant can be computed easily for analyzing occupant movements [1]. These features can be extracted after filtering the dataset. However, before features' extraction, different noise removing filters can be applied on data as per the requirements for reducing the level of sudden variations in the user movements. For our case, a median filter is applied (see Fig. 6). After executing the filter, to show a proof-of-concept application of the presented data, the movements of an occupant are analyzed by computing the step lengths and turning angles. As shown in Fig. 7, step lengths (distances between each two consecutive spatio-temporal points in meters) and turning angles (change in direction in radians from the previous point to the current point) are calculated. For computing the step lengths and turning angles, a package named ‘moveHMM’ in R studio is used [5]. For more details on the calculation of step length and turning angle see our research article [4].

**Fig. 8:**
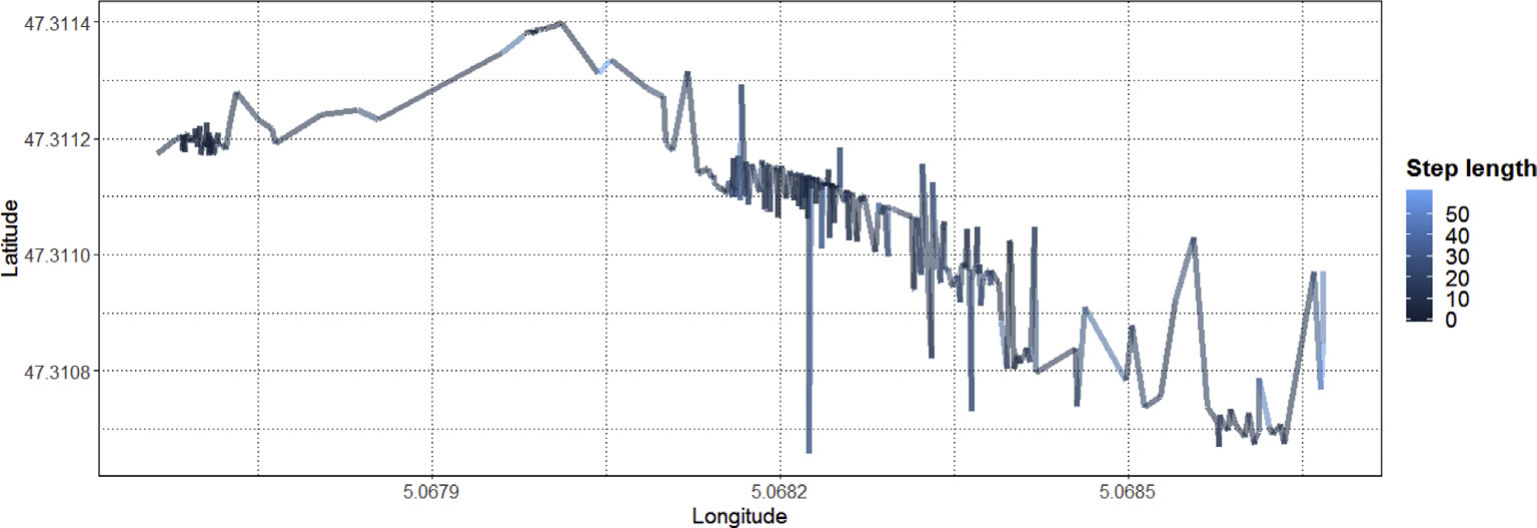
Segmenting a trajectory with the information of step length in meters.

**Fig. 9:**
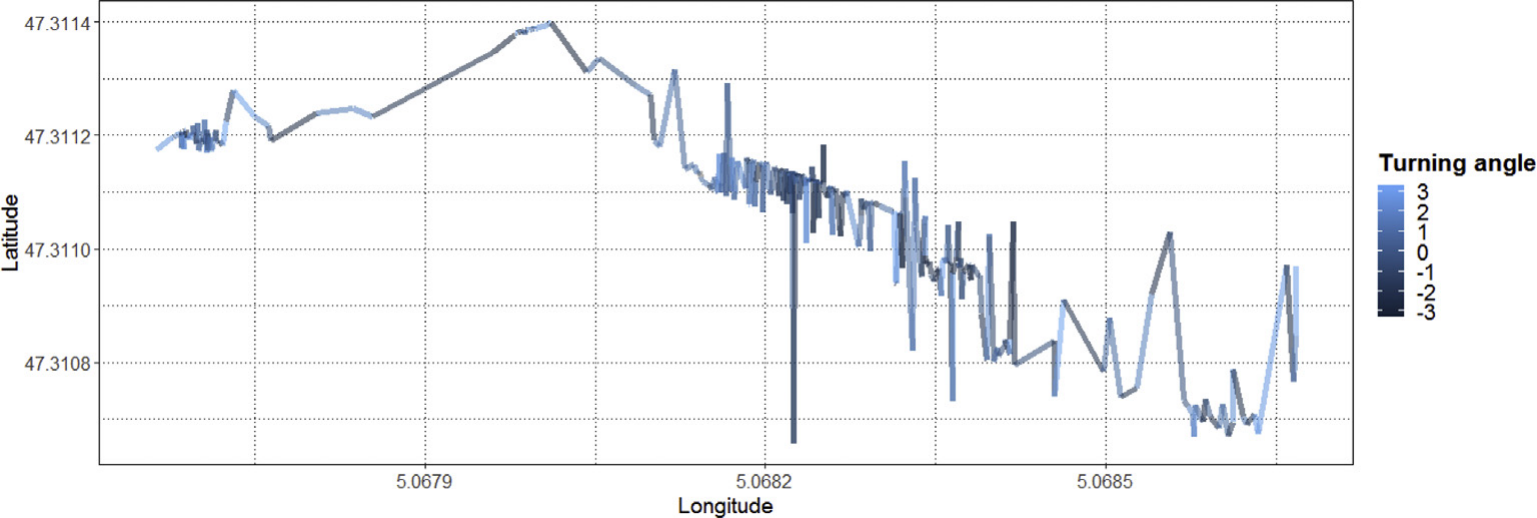
Segmenting a trajectory with the information of turning angles in radians.

The different values of step length and turning angle exists in a user trajectory which spans over 1000 spatio-temporal points are shown in Fig. 7 (top row). Whereas, Fig. 7 (bottom row) presents the frequency of different values of step length and turning angle in a trajectory. After computing the step lengths and turning angles, their values are used for segmenting a user trajectory to analyze their movements within the facility as shown in Fig. 8, Fig. 9. The segmenting a user trajectory with the information of step length and turning angle is one of the fundamental use cases which helps to analyze the movements of a user by identifying the uncertain values of step length and turning angles in a user trajectory. The resulted analyses will help us to generate stay, walk and run segments by utilizing the trajectory information. The generated stay segments will provide insights about the stay locations of users in different regions of a building [4]. Whereas, the run segments will indicate the disturbance in a building which should be considered for safety management [4]. Moreover, the extracted information can also be useful for managing building resources (e.g. energy) based on the user movements which will eventually lead to enhancement of physical comfort, the work performance of occupants and safety while keeping the building resources to the optimum [6]. For the sake of demonstrating the utility of the presented dataset, only two multifaceted characteristics (step length and turning angle) of a spatio-temporal trajectory are used. However, spatio-temporal trajectories can be pre-processed further to extract additional movement information as required [7].

The provided spatio-temporal dataset collected for developing the applications lack the building information context and require semantic enrichment processes [2]. Conventionally, semantic enrichment processes [2] utilize openly available or private data sources (e.g. geo-databases, etc.) to include application-specific geographical context in spatio-temporal data. To incorporate the semantic information of a building in the collected dataset, an OSM file of a building is provided to enrich each discrete-time triple with its corresponding building location context. Enriching spatio-temporal triples with the semantic of space (i.e. the building locations, where the data was acquired), and the semantics of time (i.e. time interval of an occupant movement with a start and a stop timestamp) give move meaning in extracting mobility-related behaviors of occupants in buildings [3,4]. Based on the extracted information from spatio-temporal movements and an OSM file of a building, two applications were developed. The developed applications [3,4] focused on; 1) acquiring the spatio-temporal data of occupants, 2) executing relevant safety risk assessment techniques on the processed spatio-temporal data, 3) generating timely alerts to occupants for triggering safety interventions to improve their personal behavior during hazard proximity conditions, and 4) disseminating the safety information as defined by the system user to building supervisors and safety managers to take quick actions for maintaining building safety.

The presented dataset is primarily used for developing the applications of safety management in dynamic environments. Analyzing the pre-processed spatio-temporal trajectories can lead to important insights about user movements in facilities. The data article presents a very basic data processing of spatio-temporal trajectories. For advanced trajectory-based applications to understand occupant behaviors, processed trajectories need to be transformed into matrixes, tensors, and graphs for performing additional computations to extract knowledge [7]. Eventually, transformed spatio-temporal data can also be used for different built environment applications such as; a) mining mobility-related patterns of occupants and predicting their next locations, b) identifying the dense regions of a building based on the number of user trajectories, c) exploring individual as well as collective behaviors by applying similarity, clustering and classification techniques for testing their developed prediction algorithms by comparing actual behaviors versus predicted behaviors to study behaviors of occupants.
